# A novel dynamic nomogram based on clinical features and laboratory indicators for diagnosis of post-neurosurgery intracranial infection

**DOI:** 10.1515/tnsci-2025-0382

**Published:** 2025-10-07

**Authors:** Minjie Tang, Qingwen Lin, Kengna Fan, Zeqin Zhang, Weiqing Zhang, Qi Wang, Tianbin Chen, Qishui Ou, Xiaofeng Liu

**Affiliations:** Department of Laboratory Medicine, Gene Diagnosis Research Center, The First Affiliated Hospital, Fujian Medical University, Fuzhou, China; Department of Laboratory Medicine, National Regional Medical Center, Binhai Campus of the First Affiliated Hospital, Fujian Medical University, Fuzhou, 350212, China; Fujian Key Laboratory of Laboratory Medicine, The First Affiliated Hospital, Fujian Medical University, Fuzhou, China; Fujian Clinical Research Center for Clinical Immunology Laboratory Test, The First Affiliated Hospital, Fujian Medical University, Fuzhou, China; Department of Laboratory Medicine, Geriatric Hospital Affiliated to Wuhan University of Science and Technology, Wuhan, 430065, China

**Keywords:** neurosurgery, intracranial infection, nomogram, diagnosis

## Abstract

**Objective:**

Intracranial infection is a serious complication after neurosurgery. However, the early diagnosis of post-neurosurgical intracranial infection (PNICI) remains challenging. The purpose of this study was to compare clinical characteristics and common laboratory indicators in patients with and without intracranial infections after neurosurgery and construct a diagnostic model of PNICI and assess its diagnostic efficacy.

**Methods:**

A total of 623 patients who underwent neurosurgery from January 2018 to October 2021 were enrolled and divided into a training set and a validation set. SPSS 22.0 software was used to compare the differences in basic information and laboratory examination results between the two groups to screen out valuable indicators. Subsequently, a nomogram for the diagnosis of PNICI was established. Then, the receiver operating characteristic (ROC) curve, calibration diagram, and decision curve analysis (DCA) were performed to evaluate the discriminative ability, consistency, and clinical usefulness of the nomogram.

**Results:**

The diagnostic model of PNICI consisted of seven variables: meningeal irritation, fever, postoperative drainage, cerebrospinal fluid (CSF) white blood cells, CSF chlorine, the CSF/blood glucose ratio, and blood neutrophil percentage. The model achieved an area under the ROC curve of 0.958 in the training set and 0.966 in the validation set. At the optimal cutoff of 0.397, the training set demonstrated 90.4% sensitivity and 90.8% specificity. The calibration curves and DCA curves of the nomogram demonstrated that the model exhibited good goodness of fit and showed a net benefit from its use.

**Conclusions:**

We developed an easily applicable nomogram using routinely available indicators. This tool enables early risk stratification for PNICI, facilitating timely interventions that may reduce infection-related complications. However, multicenter prospective validation data are required to further confirm the clinical utility.

## 1 Introduction

Post-neurosurgery intracranial infection (PNICI), with an incidence rate of approximately 0.8–19.8%, is a serious complication of neurosurgery with increased mortality, prolonged hospitalization, and higher costs [1,2]. In PNICI, bacterial infections (primarily gram-positive, though gram-negative cases are increasing) predominate, with fungal infections being rare [3,4]. Typical manifestations including fever, headache, vomiting, altered consciousness, and cerebrospinal fluid (CSF) findings including elevated white blood cells (WBC), high protein, low chloride, and low glucose, all lack specificity due to postoperative stress. Although CSF culture is the gold standard, its utility is limited by turnaround time, prior antibiotic use, and low pathogen yield.

For the early diagnosis of PNICI, previous studies have identified risk factors, including age, surgical site, duration of surgery closely related to PNICI [2,5]. But these lack sufficient diagnostic accuracy. Concurrently, some novel markers have been discovered, such as specific inflammatory factors [6] and RNA [7]. However, they have not been widely used in clinical practice and their clinical utility has yet to be further confirmed. Although emerging technologies like next-generation metagenomic sequencing demonstrate significant diagnostic value [8], their high cost and the fact that limited hospitals availability restrict routine clinical application. Consequently, combining routine indicators may improve diagnostic efficacy.

In summary, individual clinical or laboratory indicators have limited diagnostic power for PNICI. Consequently, finding an effective and feasible diagnostic method for PNICI is critical. Meanwhile, combining multiple common indicators has shown higher diagnostic efficacy than single indicators [9,10]. Therefore, we aimed to integrate common clinical and laboratory indicators into a diagnostic model for PNICI. The nomogram is a visual model that combines various diagnostic indices and enhances diagnostic efficiency, which uses several scale lines to represent different variables and calculates the individual probabilities of a clinical event based on the entire sum of variables [10]. At present, the nomogram has attracted attention in the evaluation of tumor prognosis as well as in the differential diagnosis of various diseases [10–14]. Few studies have been reported on the diagnostic value of the PNICI nomogram model that combines clinical data and laboratory indices.

Therefore, we retrospectively analyzed data from the First Affiliated Hospital of Fujian Medical University to compare patients with and without PNICI, screening variables to construct and validate a diagnostic nomogram for early PNICI detection.

## 2 Materials and methods

### 2.1 Study subjects

In this study, patients who underwent neurosurgery from January 2018 to October 2021 were enrolled according to the inclusion and exclusion criteria. Subjects were included when satisfying the following criteria: patients undergoing neurosurgery in our hospital, including urgent surgery and elective surgery, had one of the following diseases: (a) central nervous system tumors such as meningioma, glioma, acoustic neuroma, pituitary tumor, and others; (b) intracranial hematoma removal; (c) cerebrovascular diseases such as aneurysm clipping, arteriovenous malformation resection, and others; (d) others such as cyst resection, neurovascular decompression, epileptic surgery, ventriculoperitoneal shunt, etc. And subjects were excluded when satisfying the following criteria: (a) spinal surgery, (b) patients under the age of 18, (c) no postoperative cerebrospinal fluid examination, and (d) the patient was preoperatively diagnosed with an infectious disease of the central nervous system or postoperatively diagnosed with a brain abscess. The Branch for Medical Research and Clinical Technology Application, Ethics Committee of the First Affiliated Hospital of Fujian Medical University approved the study (approval no.: MRCTA, ECFAH of FMU [2021] 628). The study was conducted following the ethical standards of the 2008 Declaration of Helsinki and its later amendments. The article was prepared in accordance with Strengthening the Reporting of Observational Studies in Epidemiology.

PNICI primarily includes post-neurosurgical epidural abscess, subdural empyema, meningitis, and ventriculitis, as well as meningitis and ventriculitis associated with ventricular and lumbar cisternal drainage. The diagnostic criteria for PNICI were listed as follows referring according to Chinese expert consensus on the diagnosis and treatment of infection in neurocritical care patients (2017) and Infectious Diseases Society of America’s Clinical Practice Guidelines for Healthcare-Associated Ventriculitis and Meningitis (2017) [15,16]: (a) routine CSF parameters revealed a combined WBC count of >100 × 10^6^/L and glucose <2.6 mmol/L (or a CSF/blood glucose ratio [GluR] >0.66); (b) one or more of the following three elements: (i) persistently high body temperature (>38°C), (ii) meningeal irritation, (iii) blood leukocyte count >10^9^/L with >75% neutrophils; and (c) positive CSF bacteria culture results or the presence of bacteria validated using molecular biological techniques (excluding contamination). Meeting criteria (c) or those who did not fulfill (c) but met (a) and (b) were used to diagnose intracranial infection. PNICI diagnosis confirmation was independently performed by two neurosurgeons.

**Figure 1:**
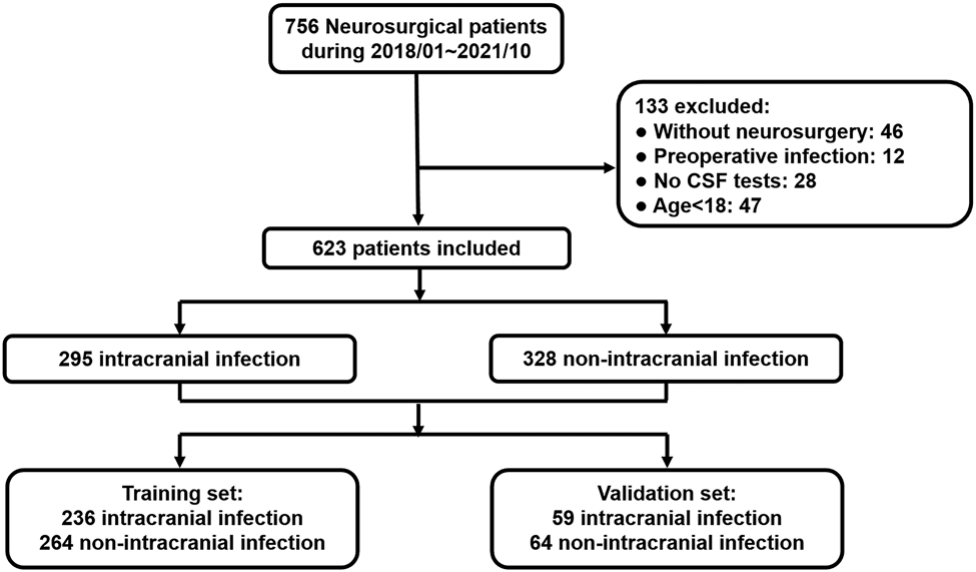
Flow chart of the present study.

Finally, 623 patients were enrolled with the detailed descriptions in Figure 1. These patients were divided into a training set and a validation set according to the time of admission. In the training set, there were 236 and 264 patients in the intracranial infection and non-intracranial infection groups, respectively. The validation set consisted of 59 patients in the intracranial infection group and 64 patients in the non-intracranial infection group.

### 2.2 Data collection

The demographic, clinical, and laboratory data were obtained from electronic medical records. To minimize assessment bias, three researchers independently extracted raw parameters while blinded to PNICI diagnostic status. They accessed only objective measurements without viewing physicians’ diagnostic notes. The detailed variables in this study covered age, gender, basic diseases (hypertension, diabetes), hospitalization time, Glasgow Coma Scale (GCS) score, prophylactic antibiotic use, emergency surgery, reasons for surgery, type of surgery, surgical site, duration of surgery, postoperative drainage, the operation frequency during hospitalization, postoperative pulmonary infection, CSF leakage, intracranial pressure, temperature, meningeal irritation, routine examination and biochemical of CSF, the CSF/blood glucose ratio, blood routine examination, C-reactive protein (CRP), and procalcitonin (PCT).

### 2.3 Statistical analysis

All statistical tests were carried out on IBM SPSS 22.0. (IBM Corp.). This study used G*Power to estimate the sample size, according to the actual situation, the loss of follow-up rate can be adjusted in the range of 5–20%. The missing values will be implemented using multiple imputation. Frequency and percentages were presented for categorical variables. And the chi-square test or Fisher’s exact test was used for comparison. Continuous variables were reported as mean with SD or median with interquartile range (IQR), and the *T*-test or the Mann–Whitney *U*-test was used to compare the intracranial infection group to the non-intracranial infection group. The training set was used to construct the models, and the validation set was used to verify the models. Candidate variables with *P* < 0.10 in univariate analyses were entered into multivariate logistic regression. Through backward stepwise selection (exit criterion *P* > 0.05), variables retaining independent predictive value were included in the final model (CSF leukocyte data were converted into a logarithmic scale of base ten for analysis). Multicollinearity was assessed through variance inflation factors (VIF) and tolerance. Variables with VIF > 5 or tolerance <0.2 would be excluded. Subsequently, the nomogram was constructed by using R4.1.0 (General Public License) according to selected variables. Finally, in the training set and validation set, the C-index, receiver operating characteristic (ROC) curve, calibration curve, and decision curve analysis (DCA) was conducted to evaluate the nomogram’s diagnostic efficacy and clinical usefulness. A two-sided *P* < 0.05 was considered statistically significant.


**Ethical approval:** The research related to human use has been complied with all the relevant national regulations, institutional policies and in accordance the tenets of the Helsinki Declaration and has been approved by the authors’ institutional review board or equivalent committee. The Branch for Medical Research and Clinical Technology Application, Ethics Committee of the First Affiliated Hospital of Fujian Medical University approved the study (approval no.: MRCTA, ECFAH of FMU [2021]628). The study was conducted following the ethical standards of the 2008 Declaration of Helsinki and its later amendments.
**Informed consent:** Informed consent has been obtained from all individuals included in this study.

## 3 Result

### 3.1 Baseline clinical characteristics

The clinical characteristics of the training and validation sets are presented in Table 1, while those of the overall study cohort are detailed in Table S1. In the training set, the intracranial infection was more likely to be male (*P* = 0.011), had a lower initial GCS (*P* = 0.017), and had longer hospitalization (*P* < 0.001). As for surgery factors, multiple operations (*P* < 0.001), posterior cranial surgery (*P* = 0.001), postoperative drainage (*P* = 0.002), and postoperative CSF leakage (*P* = 0.007) in the intracranial infection group were significantly higher than in another one. In terms of clinical characteristics, the body temperature (*P* < 0.001) of the intracranial infection group increased significantly, and the positive rate of meningeal irritation (*P* < 0.001) increased significantly.

**Table 1:** Comparison of general data and clinical features between patients with and without intracranial infection after neurosurgery

Variable	Training set			Validation set		
	Intracranial infection (*n* = 236)	Non-intracranial infection (*n* = 264)	*P* value	Intracranial infection (*n* = 59)	Non-intracranial infection (*n* = 64)	*P* value
Male (*n*, %)	150 (63.6%)	138 (52.3%)	0.011	32 (54.2%)	35 (54.7%)	0.960
Age (IQR, y)	52 (41, 61)	54 (43, 62)	0.357	48 (36, 58)	58 (44, 64)	0.016
Hypertension (*n*, %)	47 (21.2%)	77 (29.3%)	0.041	16 (27.1%)	20 (31.3%)	0.615
Diabetes (*n*, %)	19 (8.6%)	32 (12.2%)	0.197	8 (13.6%)	5 (7.8)	0.300
Pneumonia (*n*, %)	72 (32.4%)	69 (26.2%)	0.134	9 (15.3%)	12 (18.8%)	0.607
CSF leakage (*n*, %)	6 (2.7%)	0 (0%)	0.007	4 (6.8%)	0 (0%)	0.034
Hospitalization time (IQR, d)	24 (17, 33)	16 (12, 22)	<0.001	26 (17, 33)	16 (11, 22)	<0.001
Fever (IQR, °C)	38.5 (38.0, 39.0)	37.6 (37.0, 38.3)	<0.001	38.4 (38.0, 39.0)	37.5 (36.9, 38.1)	<0.001
Meningeal irritation (*n*, %)	102 (43.2%)	17 (6.4%)	<0.001	40 (67.8%)	1 (1.6%)	<0.001
ICP (IQR, mmHg)	180 (145, 260)	175 (130, 235)	0.140	210 (175, 300)	175 (150, 240)	0.049
Initial GCS	15 (14, 15)	15 (15, 15)	0.017	15 (15, 15)	15 (15, 15)	0.289
Emergency surgery (*n*, %)	67 (30.3%)	67 (25.9%)	0.279	20 (31.3%)	12 (20.3%)	0.168
Multiple operations (*n*, %)	45 (19.2%)	18 (6.9%)	<0.001	9 (15.3%)	2 (3.1%)	0.019
Surgical duration (IQR, h)	3.75 (2.5, 5)	3.66 (2.5, 4.66)	0.323	4.21 (2.83, 5.00)	3.50 (2.37, 4.46)	0.058
Drainage (*n*, %)	87 (37%)	64 (24.3%)	0.002	22 (37.3%)	17 (26.6%)	0.202
Antibiotic prophylaxis (*n*, %)	100 (42.4%)	105 (39.8%)	0.555	30 (50.8%)	25 (39.1%)	0.189
Posterior fossa surgery (*n*, %)	70 (29.8%)	45 (17.1%)	0.001	21 (35.6%)	13 (20.3%)	0.058
**Reason for surgery (** ***n*** **, %)**			0.018			0.148
Tumor	106 (45.1%)	115 (43.7%)		35 (59.3%)	25 (39.1%)	
Vascular	50 (21.3%)	84 (31.9%)		14 (23.7%)	21 (32.8%)	
Hematoma	34 (14.5%)	22 (8.4%)		2 (3.4%)	5 (7.8%)	
Others	45 (19.1%)	42 (16%)		8 (13.6%)	13 (20.3%)	
**Surgery types (** ***n*** **, %)**			0.069			0.102
Craniotomy	189 (80.1%)	192 (72.7%)		50 (847%)	43 (67.2%)	
EET	16 (6.8%)	22 (8.3%)		1 (1.7%)	3 (4.7%)	
Via femoral artery puncture	10 (4.2%)	29 (11%)		4 (6.8%)	13 (20.3%)	
Others	21 (8.5%)	21 (7.6%)		4 (6.8%)	5 (7.8%)	

*n*: Number; IQR: interquartile range; *P*: comparison between the intracranial infection group and the non-intracranial infection group; GCS: Glasgow Coma Scale; CSF: cerebrospinal fluid; ICP: intracranial pressure; EET: endoscopic endonasal transsphenoidal surgery.

### 3.2 Differences in laboratory findings

Meanwhile, comparative analysis of laboratory profiles revealed significant intergroup differences (Table 2). The intracranial infection group exhibited elevated hematological indices: CRP (*P* < 0.001), blood WBC (*P* < 0.001), neutrophils (*P* < 0.001), lymphocytes (*P* < 0.001), monocytes (*P* = 0.019), eosinophils (*P* = 0.013), basophils (*P* = 0.042), and hemoglobin distribution width (*P* = 0.014). CSF parameters: leukocyte count, protein, lactate, erythrocyte count, and polymorphonuclear cell percentage (all *P* < 0.001). Conversely, this group demonstrated reduced: CSF glucose, chloride, and CSF-to-blood glucose ratio (all *P* < 0.001).

**Table 2:** Comparison of laboratory index between patients with and without intracranial infection after neurosurgery

Variable	Training set			Validation set		
	PNICI (*n* = 236)	Non-PNICI (*n* = 264)	*P* value	PNICI (*n* = 59)	Non-PNICI (*n* = 64)	*P* value
**Peripheral blood parameters (IQR)**						
CRP (mg/L)	48.53 (22.00, 87.00)	31.94 (11.79, 62.99)	<0.001	41.70 (31.5, 78.5)	31.35 (9.05, 64.84)	0.002
PCT (ng/mL)	0.07 (0.04, 0.17)	0.05 (0.04.0.13)	0.06	0.07 (0.05, 0.11)	0.07 (0.05, 0.11)	0.756
WBC (10^9^/L)	11.42 (8.76, 14.62)	9.94 (7.66, 12.67)	<0.001	11.69 (9.26, 15.63)	10.40 (7.70, 12.92)	0.023
NEU (%)	83.2 (77.5, 87.4)	77.9 (71.3, 84.2)	<0.001	82.1 (76.6, 87.2)	78.6 (71.9, 85.8)	0.041
NEU (10^9^/L)	9.59 (7.02, 12.75)	7.64 (5.54, 10.05)	<0.001	9.10 (7.01, 12.33)	7.44 (5.97, 9.93)	0.037
LYM (%)	9.2 (5.9, 13.4)	12.9 (8.6, 18.6)	<0.001	9.4 (6.0, 15.7)	12.7 (8.3, 17.8)	0.032
LYM (10^9^/L)	1.02 (0.7, 1.35)	1.22 (0.95, 1.80)	<0.001	1.11 (0.65, 1.67)	1.18 (0.79, 1.78)	0.362
MON (%)	5.6 (4.18, 7.13)	6.0 (4.3, 7.4)	0.610	5.3 (4.2, 7.15)	5.7 (4.4, 7.4)	0.643
MON (10^9^/L)	0.62 (0.44, 0.81)	0.57 (0.37, 0.80)	0.019	0.56 (0.40, 0.96)	0.60 (0.39, 0.83)	0.557
EOS (%)	0.3 (0.1, 0.9)	0.6 (0.2, 1.1)	<0.001	0.3 (0.2, 0.9)	0.5 (0.2, 0.9)	0.139
EOS (10^9^/L)	0.03 (0.02, 0.09)	0.06 (0.02, 0.1)	0.013	0.04 (0.02, 0.08)	0.05 (0.03, 0.11)	0.265
BAS (%)	0.2 (0.1, 0.3)	0.2 (0.1, 0.3)	<0.001	0.2 (0.1, 0.3)	0.2 (0.1, 0.3)	0.505
BAS (10^9^/L)	0.02 (0.01, 0.03)	0.02 (0.01, 0.04)	0.042	0.02 (0.01, 0.04)	0.02 (0.01, 0.03)	0.910
LUC (%)	1.1 (0.7, 1.6)	1.2 (0.8, 1.7)	0.172	0.9 (0.5, 1.45)	1.1 (0.6, 1.5)	0.386
LUC (10^9^/L)	0.12 (0.08, 0.18)	0.11 (0.08, 0.17)	0.455	0.10 (0.06, 0.17)	0.11 (0.07, 0.15)	0.844
RBC (10^12^/L)	3.78 (3.29, 4.16)	3.85 (3.41, 4.26)	0.101	3.73 (3.36, 4.13)	3.83 (3.54, 4.34)	0.141
MCV (fL)	89.5 (86.0, 92.8)	89.5 (86.6, 92.4)	0.938	90.0 (86.3, 93.1)	91.1 (86.9, 93.7)	0.204
MCH (pg)	30.7 (29.7, 31.7)	30.4 (29.5, 31.4)	0.225	30.4 (28.9, 31.5)	30.7 (29.4, 31.8)	0.469
MCHC (g/L)	343 (333, 351)	341 (332, 351)	0.554	335 (324, 344)	335 (327, 347)	0.593
RDW (%)	13.6 (12.8, 14.4)	13.5 (12.7, 14.2)	0.298	13.8 (13.1, 14.7)	13.6 (12.9, 14.2)	0.142
HDW (g/L)	25.2 (23.5, 27.6)	24.5 (22.6, 26.9)	0.014	25.3 (23.1, 27.6)	24.6 (22.7, 26.9)	0.198
PLT (10^12^/L)	230 (190, 303)	231 (191, 293)	0.629	228 (176, 300)	224 (177, 271)	0.555
MPV (fL)	8.2 (7.7, 9.1)	8.3 (7.5, 9.2)	0.810	7.9 (7.5, 9.2)	8.0 (7.4, 8.8)	0.949
PDW (%)	53.1 (47.3, 58.9)	52.3 (47.0, 59.2)	0.631	52.4 (48.2, 59.2)	51.2 (46.7, 56.9)	0.544
MPC (g/L)	268 (249, 281)	267 (252, 282.5)	0.618	270 (246, 281)	271 (257, 281)	0.474
**CSF parameters (IQR)**						
WBC (10^6^/L)	1,339 (495, 2,945)	62 (14, 227)	<0.001	1,950 (631, 2,754)	61 (18, 282)	<0.001
Glucose (mmol/L)	2.27 (1.65, 3.2)	3.25 (2.67, 4.11)	<0.001	2.50 (1.77, 3.71)	3.42 (2.66, 4.32)	<0.001
GluR (%)	39.66 (28.56, 50.88)	59.80 (48.82, 73.31)	<0.001	39.71 (27.85, 50.28)	60.45 (39.45, 72.95)	<0.001
Protein (g/L)	2.06 (1.39, 3.00)	0.88 (0.56, 1.58)	<0.001	2.05 (1.42, 3.00)	0.93 (0.47, 1.80)	<0.001
Chloride (mmol/L)	116 (112, 120)	118 (115, 123)	<0.001	116 (112, 118)	119 (116, 121)	<0.001
Lactate (mmol/L)	5.10 (3.66, 7.11)	2.60 (2.00, 3.67)	<0.001	4.60 (3.70, 6.35)	3.25 (2, 4.35)	<0.001
RBC (10^9^/L)	6.6 (1.0, 38)	1.8 (0.2, 11.2)	<0.001	10.5 (1.2, 59.2)	5.3 (0.3, 23.2)	0.031
Multinuclear leukocytes (%)	79.4 (70.7, 86.1)	48.3 (18.1, 72.9)	<0.001	75.7 (63.6, 83.8)	50.0 (26.0, 72.5)	<0.001

CRP: C-reactive protein; PCT: procalcitonin; WBC: white blood cell; NEU: neutrophil; LYM: lymphocyte; MON: monocyte; EOS: eosinophil; BAS: basophil; LUC: large unstained cell; RBC: red blood cell; Hb: hemoglobin; HCT: hematocrit; MCV: mean corpuscular volume; MCH: mean RBC hemoglobin content; MCHC: mean RBC hemoglobin concentration; RDW: red cell distribution width; HDW: hemoglobin distribution width; PLT: platelet; MPV: mean platelet volume; PDW: platelet distribution width; MPC: mean platelet content concentration; GluR: the CSF/blood glucose ratio.

### 3.3 Development of the diagnostic nomogram model

The differential variables from the univariate analysis were analyzed by multivariate logistic regression (considering that the hospitalization time was only recorded at discharge, it was excluded for further analysis). The results showed that fever (OR = 3.474, [95% confidence interval (CI), 2.105–5.732], *P* < 0.001), meningeal irritation (OR = 9.804, [95% CI, 3.455–27.822], *P* < 0.001), postoperative drainage (OR = 5.089, [95% CI, 2.223–11.651], *P* < 0.001), CSF WBC (OR = 11.559, [95% CI, 6.138–21.77], *P* < 0.001), CSF chloride (OR = 0.94, [95% CI, 0.892–0.99], *P* = 0.019), the CSF/blood glucose ratio (OR = 0.948, [95% CI, 0.928–0.969], *P* < 0.001), and blood neutrophil percentage (OR = 1.07, [95% CI, 1.027–1.115], *P* < 0.001) were diagnosis indexes associated with PNICI (Table 3). All predictors demonstrated excellent independence with VIF ranging from 1.06 to 1.50, well below the critical threshold of 5. Tolerance values exceeded 0.6 in all cases, confirming minimal collinearity risk (Table S2).

**Table 3:** Multivariate logistic regression analysis for PNICI

Variable	*B*	SE	Wald	df	*P*	OR	95% CI
Fever	1.245	0.256	23.747	1	<0.001	3.474	2.105–5.732
Meningeal irritation	2.283	0.532	18.399	1	<0.001	9.804	3.455–27.822
Drainage	1.627	0.423	14.82	1	<0.001	5.089	2.223–11.651
GluR	−0.053	0.011	22.868	1	<0.001	0.948	0.928–0.969
CSF-chloride	−0.062	0.026	5.523	1	0.019	0.94	0.892–0.99
B-NEU%	0.067	0.021	10.328	1	<0.001	1.07	1.027–1.115
CSF-WBC	2.448	0.323	57.422	1	<0.001	11.559	6.138–21.77
Intercept	−49.478	9.556	26.811	1	<0.001	0	

B-NEU%: blood neutrophil proportions; GluR: the CSF/blood glucose ratio; CSF_WBC: CSF white blood cells.

**Figure 2:**
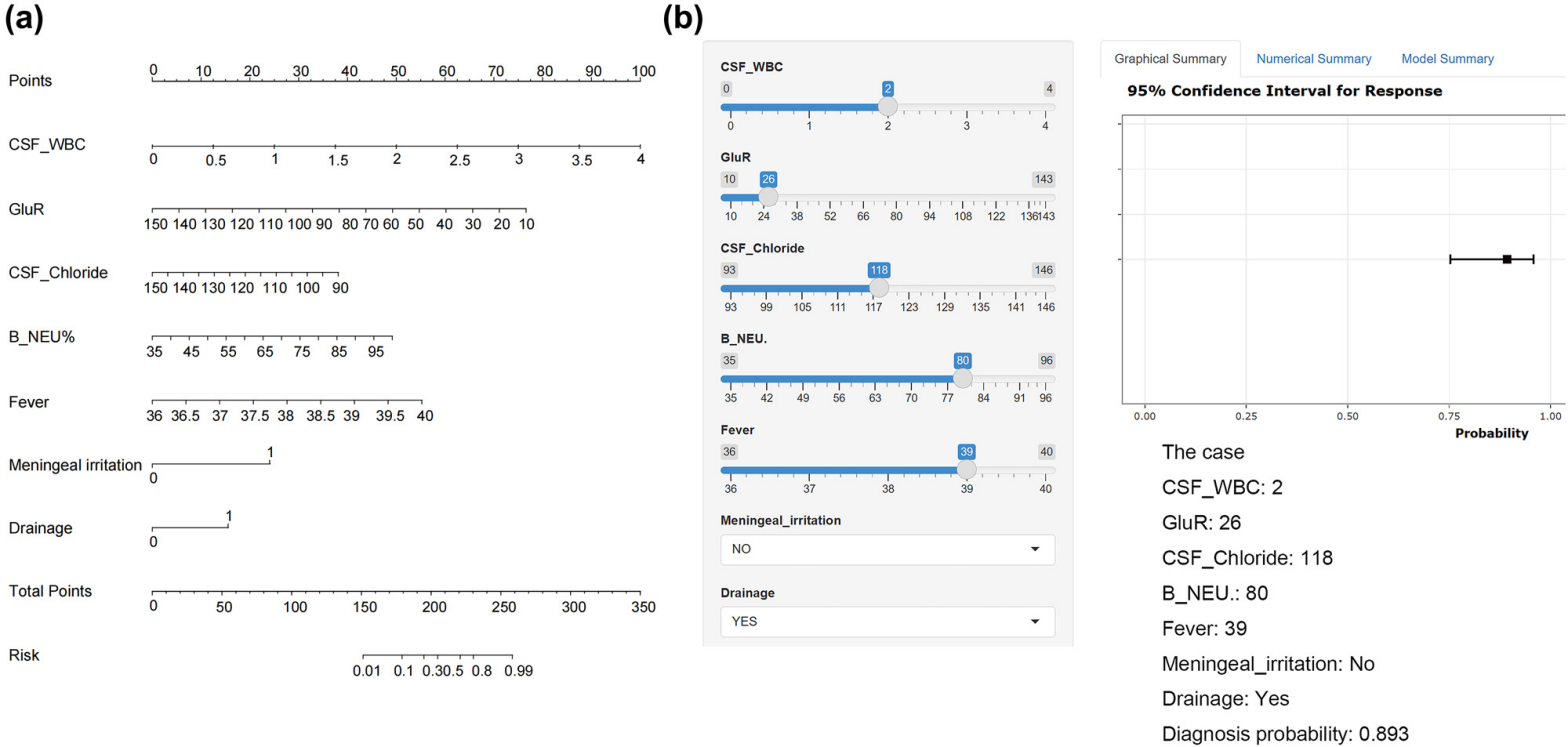
Nomogram for the diagnosis of PNICI. Draw vertical lines from each variable value to the “Points” axis. Subsequently, sum all points to obtain total points. Align total points with the probability axis to determine individual PNICI risk. Defining categorical variables, 0 = no, 1 = yes. (a) The nomogram of PNICI and (b) the web-based dynamic nomogram (https://nomogram-pnici.shinyapps.io/DynNomPNICI/). CSF_WBC: CSF white blood cells; GluR: the CSF/blood glucose ratio; B_NEU%: blood neutrophil proportions.

These seven variables were brought into the R4.1.0 software and used the rms package to construct the nomogram. The nomogram showed the respective scores of the 7 variables and the diagnosis probability of PNICI corresponding to the total score obtained by adding the scores of each variable. The higher the total score, the higher the likelihood of being diagnosed with PNICI (Figure 2a). Meanwhile, as shown in Figure 2b, the dynamic nomogram was developed which can be available online (https://nomogram-pnici.shinyapps.io/DynNomPNICI/) to facilitate clinical implementation. For example, in a 45-year-old patient with fever (39°C) and post-drainage status, inputting laboratory values – including CSF WBC (100 × 10^6^/L), CSF chloride (118 mmol/L), blood neutrophils (80%), and CSF glucose ratio (26%) – into the online tool automatically calculates a bacterial meningitis probability of 89.3%. This high-risk prediction would indicate immediate empirical antibiotic initiation while awaiting culture results. The nomogram thus transforms complex data into actionable clinical decisions within seconds.

### 3.4 Comparative diagnostic performance

Furthermore, we compared the diagnostic performance of the nomogram and CSF culture for PNICI in the training set (Tables S3 and S4). As shown in Table 4, the nomogram demonstrated markedly higher sensitivity than CSF culture (92.0% [95% CI: 88.1–94.8] vs 28.0% [22.5–34.2], *P* < 0.001), reducing missed diagnoses by 64.0%. Crucially, it detected 92.9% (158/170) of culture-negative infections. While CSF culture showed higher specificity (98.9 vs 93.9%, *P* = 0.012), its 72% false-negative rate (170/236 infections missed) limits clinical utility as a screening tool. The nomogram’s high NPV (92.9 vs 60.6%, *P* < 0.001) further supports its role for rapid exclusion of infection.

**Table 4:** Diagnostic performance comparison in training set: nomogram vs CSF culture (*n* = 500)

Metric	Nomogram model (95% CI)	CSF culture (95% CI)	*P*-value
Sensitivity	92.0% (88.1–94.8)	28.0% (22.5–34.2)	<0.001
Specificity	93.9% (90.6–96.3)	98.9% (96.8–99.7)	0.012
Positive predictive value	93.1% (89.9–96.4)	95.7% (90.8–100)	0.421
Negative predictive value)	92.9% (89.8–96.0)	60.6% (56.0–65.2)	<0.001
Positive likelihood ratio (LR+)	15.1 (9.8–23.2)	25.2 (8.1–78.5)	0.317
Negative likelihood ratio (LR−)	0.09 (0.06–0.13)	0.73 (0.67–0.79)	<0.001
Missed diagnosis rate	8.0% (5.2–11.5)	72.0% (65.8–77.5)	<0.001
False alarm rate	6.1% (3.7–9.4)	1.1% (0.3–3.2)	0.012
Detection rate of culture-negative infections	92.9% (88.7–96.0)	—	

CI = confidence interval; Reference standard: Final clinical diagnosis.

### 3.5 Validation of the diagnostic nomogram model

**Figure 3:**
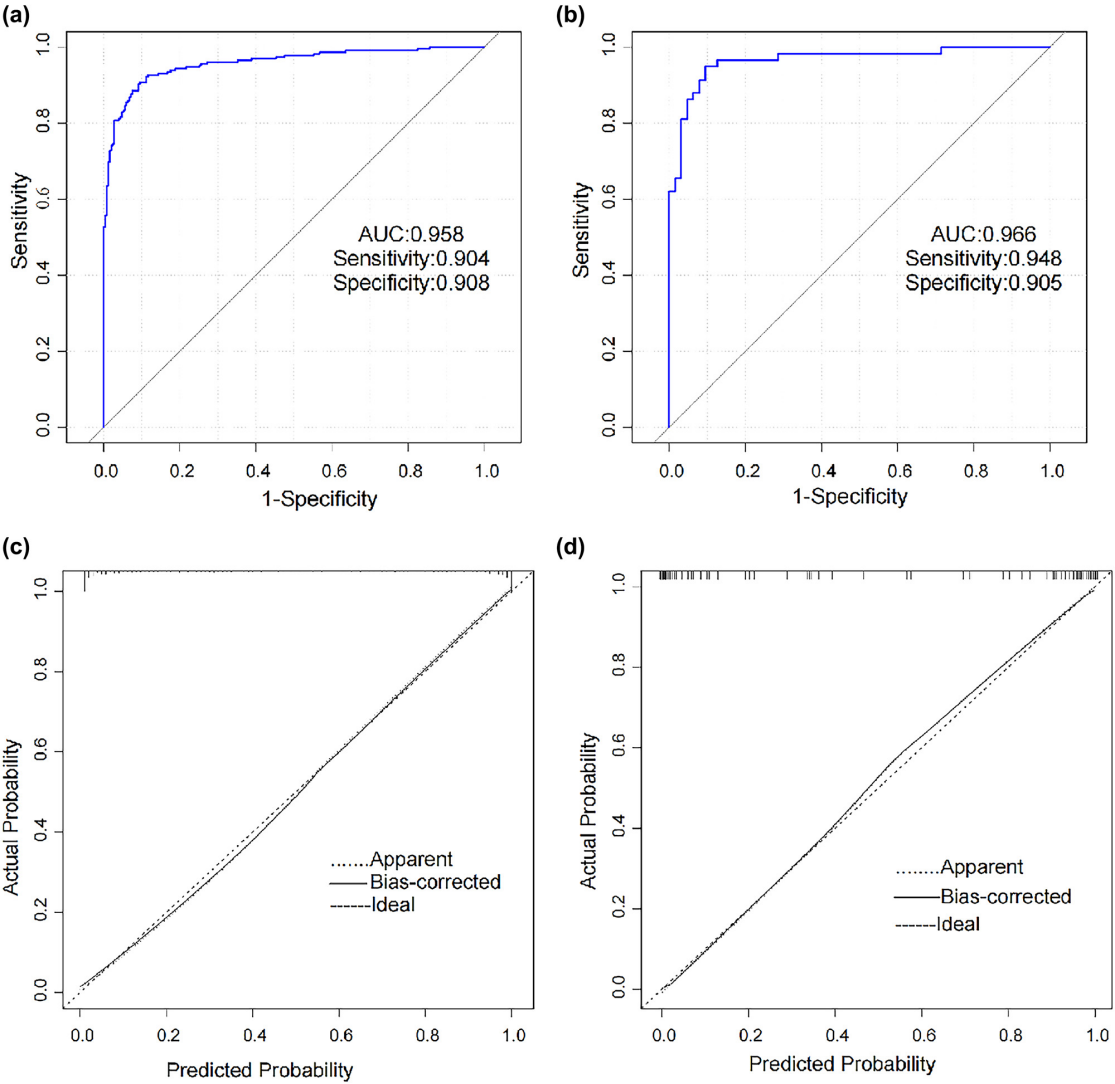
Verification of the diagnostic efficiency of the PNICI model. (a) and (b) are the ROC curve of the model. (a) ROC curve of the training set, the area under the ROC curve (AUC) was 0.958 (sensitivity 90.4%, specificity 90.8%). (b) ROC curve of the validation set, the AUC was 0.966 (sensitivity 94.8%, specificity 90.5%). (c) and (d) are the calibration curve of the model. The observed PNICI is plotted on the *y*-axis and the probability of PNICI measured by nomogram is plotted on the *x*-axis. The dotted line represents the apparent curve. The solid line is calculated by bootstrapping (resample: 1,000) and represents the discrimination of PNICI using a nomogram, and the diagonal dashed line represents the ideal line showing the diagnostic probability is consistent with the observed probability. (c) Training set’s calibration curve, mean absolute error = 0.009. (d) Validation set’s calibration curve, mean absolute error = 0.006.

Then, the ROC curves, calibration curves, and DCA curves of the training set and validation set were drawn. The nomogram model displayed powerful diagnostic ability in both the training set and the validation set, with C-indexes of 0.958 (95% CI = 0.940–0.976) and 0.966 (95% CI = 0.934–0.996). The ROC curve (Figure 3a and b) showed that at the optimal cutoff of 0.397, the sensitivity and specificity of the training set were 90.4 and 90.8%, and in the validation set, they were relatively 94.8 and 90.5%. With 1,000 cycles of bootstrap resampling, the calibration curves of the nomogram showed the solid line and the diagonal dashed line were close and partly coincided, i.e., the diagnosis probability is highly consistent with the observation probability (Figure 3c and d).

**Figure 4:**
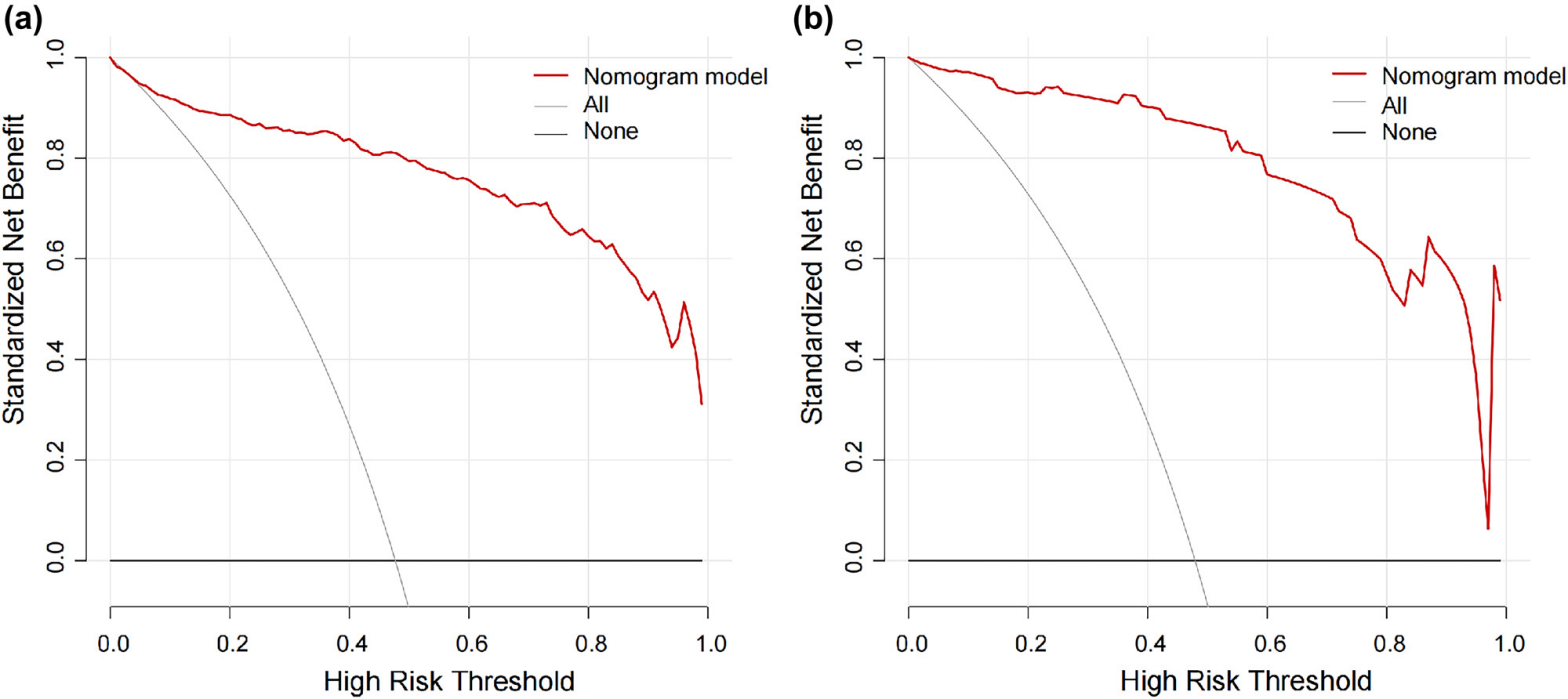
Decision curve analysis of the nomogram for PNICI. The *y*-axis represents the net benefit and the *x*-axis indicates the threshold probability. The red solid line represents the net benefit of the model. The gray line represents the assumption that all patients are PNICI. The black line represents the assumption that no patients are PNICI. (a) Training set’s DCA curve and (b) validation set’s DCA curve.

Additionally, DCA curves were used to further assess the clinical application values of the nomogram model in PNICI. As shown in Figure 4, both in the training set and validation set, when the threshold probability was between 0.1 and 99%, using the nomogram to diagnose PNICI presented a greater net benefit than using the treat-all-patients scheme or the treat-none scheme (Figure 4a and b).

## 4 Discussion

Although neurosurgery has advanced and reliable technology, complications such as intracranial infection currently seem to be an inevitable obstacle. As a serious complication after neurosurgery, PNICI poses a significant threat to the survival and recovery of patients. Even though early diagnosis is the key to improving the prognosis of PNICI patients, it remains challenging to achieve.

In this study, we provided a new diagnostic strategy for PNICI, which try to combine clinical and laboratory indicators to construct a model in the hope of providing more help for the diagnosis of PNICI. After collecting data, screening variables, and building models, the model has demonstrated favorable discrimination in our cohort. As a developmental study for a novel diagnostic tool, our primary goal was to establish proof-of-concept and internal validity under controlled conditions. Further validation studies are now the critical next step.

Fever and meningeal irritation are common clinical manifestations of intracranial infection. However, in neurosurgery, early fever frequently reflects surgical stress responses (tissue trauma, perfusion shifts) or device-related inflammation rather than true infection, complicating diagnostic triage [17,18]. Although estimating the existence of PNICI from fever alone is challenging, the severity of fever in individuals with and without intracranial infection is likely to differ. In this study, body temperature increased more obviously in intracranial infection. This is probably because the intracranial infection group has a storm of inflammatory factors caused by an infection in addition to surgical stress responses under the same conditions, so we considered the degree of elevated body temperature still contributed to the diagnosis of PNICI [16–19]. In our diagnostic model, the higher the body temperature, the higher the model’s score, and the more likely they were to get an infection.

Meningeal irritation signs are mainly caused by infection, hemorrhagic CSF, cancerous CSF, and meningeal lesions. Our results showed meningeal irritation was only 6.4% in patients without PNICI and 43.2% in patients with PNICI. When excluding meningeal stimulation caused by subarachnoid hemorrhage and tumor factors, the occurrence of postoperative meningeal irritation is valuable for the assessment of intracranial infection, especially bacterial meningitis [20,21].

Drainage tubes facilitate bacterial translocation by breaching the dura and providing a surface for biofilm formation. Our results showed that postoperative drainage was one of the diagnostic factors of PNICI, which is consistent with the results of other studies [5,20]. Meanwhile, previous research has linked the duration of drainage to PNICI, with 3 days or more of drainage time frequently being the cut-off point [1,20]. The longer the indwelling time, the easier the bacteria proliferate, and the more frequent PNICI becomes [1,16]. Removing the tube as soon as possible is beneficial to reduce the risk of infection. Meanwhile, unlike prior studies [22], surgical factors did not predict PNICI here. This may reflect our institution’s stringent infection control protocols, including mandatory antibiotic prophylaxis for all craniotomies and real-time sterilization audits, potentially neutralizing variables typically associated with infection risk.

CSF examination is an important tool for the diagnosis of PNICI. In the nomogram diagnostic model, the CSF WBC, CSF chloride, and CSF/blood glucose ratio were closely related to the diagnosis of PNICI. The elevated CSF WBC and the consumption of CSF chloride and CSF glucose during intracranial infection are the main laboratory indicators for the diagnosis of intracranial infection. However, because it is primarily delivered by blood glucose and impacted by blood glucose changes, CSF glucose levels may not accurately mirror those in the brain. Hence, the CSF/blood glucose ratio can avoid interference from blood glucose and better reflect the consumption of CSF glucose by bacteria and WBCs during intracranial infection [23,24]. This finding is consistent with this study.

Besides the CSF examination, the percentage of neutrophils in the blood was found to be a diagnostic indicator of PNICI. Multiple blood routine indicators such as WBC, neutrophils, neutrophil percentage, platelets, and other indicators are common inflammatory indicators. Although intracranial infection is mainly localized and the diagnostic efficacy of hematologic alteration has not been comprehensively cleared, some studies have shown that the WBC and the percentage of neutrophils in the blood have diagnostic value for postoperative intracranial infection [25], which agree with the results of this study.

The indicators in our nomogram reflect key pathophysiological processes in PNICI. First, postoperative drainage tubes create a physical conduit for skin flora to invade the intracranial space, especially when local immunity is compromised by surgical trauma. Subsequently, elevated body temperature and increased blood neutrophil percentage may be caused by pro-inflammatory cytokines released from infected meninges stimulate hypothalamic thermoregulation and bone marrow granulopoiesis. Meanwhile, elevated CSF WBC further exacerbates blood–brain barrier disruption via matrix metalloproteinases [26] and reactive oxygen species [27], facilitating bacterial translocation. Furthermore, the glycolytic consumption of bacteria leads to a decrease in glucose in cerebrospinal fluid. These mechanisms collectively explain why combining clinical and laboratory parameters provides superior diagnostic power compared to isolated indicators.

In contrast to previous studies that focused on PNICI risk factors or looked for diagnostic indicators, this study incorporated clinical characteristics and common laboratory indicators to screen significant factors and construct a model in order to obtain a more comprehensive and objective model. As we know, CSF indexes are crucially important for the diagnosis of postoperative intracranial infection, but the sensitivity and specificity of a single index for diagnosis are poor, and the combination of multiple indexes can increase diagnostic efficacy [23,28]. Zheng et al. and Zhai et al. built algorithms based on CSF indicators [23,28]. And their results both indicate that the multi-index algorithm is better than the single index. However, the models by Zheng and Zhai et al. relied solely on laboratory indicators, while Cheng et al.’s study on postoperative intracranial infections only compared clinical factors with albumin [29]. Furthermore, Wei et al. [30] and Yang et al.’s [31] research did not incorporate CSF parameters essential for intracranial infection diagnosis and exclusively focused on aneurysms despite the diversity of neurosurgical diseases. In contrast, this study covers diverse pathologies and integrates both clinical factors and laboratory indicators, providing a more comprehensive risk assessment.

Meanwhile, we visualize the model via the nomogram. The nomogram is currently widely used in the diagnosis, treatment, and prognosis prediction of diseases, making the model visual, simple, and understandable, and allowing for the individualized evaluation of patients [32–34].

## 5 Limitations

At the same time, there are some shortcomings in this study. First, as a single-center study, our cohort may not fully represent broader populations or healthcare settings. While this design ensured rigorous data control for nomogram development, external multi-center validation is essential to assess its generalizability and clinical applicability. Second, the types of neurosurgery included in this study included tumors, hematoma removal, cerebrovascular diseases, and other categories. If more accurate subtype classification research could be carried out for various types of cases, it may have more clinical application value. Meanwhile, although we employed multiple imputation to address missing data, this approach may not fully mitigate selection bias. Critically ill patients with incomplete records – who were consequently excluded – often represent higher-risk populations. Their underrepresentation could potentially skew model performance estimates and limit generalizability to the most severe cases. Future prospective studies should prioritize comprehensive data collection in critical care settings to validate these findings. Third, the retrospective nature of this study limits causal inference and may introduce selection bias. Future prospective cohort studies are warranted to validate the predictive performance and clinical utility of this nomogram. Nevertheless, this study is still noteworthy because it is based on clinical characteristics and laboratory results to diagnose PNICI. And in subsequent work, we will focus on the collection of external data for further validation of the model to clinicians and patients can achieve more benefits. Future efforts will focus on refining this model and implementing its integration within electronic medical records to support early clinical diagnosis and intervention.

## 6 Conclusions

This single-center study retrospectively analyzed the data of 623 patients to develop and internally validated a clinically useful nomogram with fever, meningeal irritation, postoperative drainage, CSF WBC, CSF chloride, the CSF/blood glucose ratio, and blood neutrophil percentage. This tool enables early risk stratification for PNICI, facilitating timely interventions that may reduce infection-related complications. While its implementation requires external prospective validation in multicenter cohorts to confirm generalizability before integration into electronic health record systems for real-time clinical decision support.

## Supplementary Material

Supplementary Table
