# Supplementary material for: A novel dynamic nomogram based on clinical features and laboratory indicators for diagnosis of post-neurosurgery intracranial infection
Source: Transl Neurosci. 2025 Oct 7;16(1):20250382. doi: 10.1515/tnsci-2025-0382 (PMC12514682; doi:10.1515/tnsci-2025-0382)
Supplement: Supplementary Table [file tnsci-2025-0382-sm.pdf]

Supplementary material

Table S1: Comparison of general data and clinical features between Infected patients with non-infected patients after neurosurgery

Variable	Intracranial infection (*n* = 295)	Non-intracranial infection (n=328)	*P* value
Male (*n*, %)	182(61.6%)	173(52.7%)	0.023
Age (IQR, y)	51(40,61)	55(44,62)	0.053
Hypertension (*n*, %)	63(21.3%)	97(27.5%)	0.019
Diabetes (*n*, %)	27(9.15%)	37(11.3%)	0.382
Pneumonia (*n*, %)	81(27.4%)	81(24.7%)	0.432
CSF leakage (*n*, %)	10(3.4%)	0(0%)	0.0008
Hospitalization time (IQR, d)	24(17,33)	16(12,22)	<0.0001
Fever (IQR, °C)	38.5(38,39)	37.6(37,38.2)	<0.0001
Meningeal irritation (*n*, %)	142(48.1%)	18(5.5%)	<0.0001
ICP (IQR, mmHg)	190(150,260)	172(140,235)	0.0144
Initial GCS	15(15,15)	15(15,15)	0.080
Emergency surgery (*n*, %)	87(29.5%)	79(24.1%)	0.127
Multiple operations (*n*, %)	54(18.3%)	20(6.1%)	<0.0001
Surgical duration (IQR, h)	4(2.62,5)	3.5(2.5,4.5)	0.0198
Drainage (*n*, %)	109(36.9%)	81(24.7%)	0.0009
Antibiotic prophylaxis (*n*, %)	130(44.1%)	130(39.6%)	0.262
Posterior fossa surgery (*n*, %)	91(30.8%)	58(17.7%)	0.0001
**Reason for surgery (*n*, %)**			0.023
Tumour	141(47.8%)	140(42.7%)	
Vascular	64(21.7%)	105(32.0%)	
Hematoma	36(12.3%)	27(8.23%)	
Others	53(17.9%)	55(16.8%)	
**Surgery types (*n*, %)**			0.003
Craniotomy	239(81.0%)	235(71.6%)	
EET	17(5.76%)	25(7.62%)	
Via femoral artery puncture	14(4.74%)	42(12.8%)	
Others	25(8.47%)	26(7.92%)	

Table S2: Multicollinearity diagnostics of predictors

Predictor	VIF	Tolerance
logWBC	1.50	0.667
Glucose ratio	1.28	0.779
CSF chloride	1.12	0.890
Blood neutrophils	1.09	0.915
Fever	1.20	0.836
Meningeal irritation	1.17	0.857
Postoperative drainage	1.06	0.941

Table S3: Confusion matrix: Nomogram model vs Final diagnosis

	Final diagnosis positive	Final diagnosis negative
Nomogram positive	217	16
Nomogram negative	19	248

Table S4: Confusion matrix: CSF culture vs Final diagnosis

	Final diagnosis positive	Final diagnosis negative
Culture positive	66	3
Culture negative	170	261
